# A Narrative Review on Pain Management in Head and Neck Cancer: Integrating Multimodal Analgesia and Interventional Procedures

**DOI:** 10.5812/aapm-146825

**Published:** 2024-06-01

**Authors:** Farnad Imani, Saleh Mohebbi, Masood Mohseni, Behnaz Karimi, Saeid Rahimi, Gholam Ali Dikafraz Shokooh

**Affiliations:** 1 Pain Research Center, School of Medicine, Iran University of Medical Sciences, Tehran, Iran; 2 Skull Base Research Center, the Five Senses Health Institute, School of Medicine, Iran University of Medical Sciences, Tehran, Iran

**Keywords:** Neck Pain, Cancer Pain, Pain Management, Chronic Pain, Head and Neck Neoplasms

## Abstract

Surgical interventions and radiotherapy for head and neck cancer frequently result in substantial instances of acute and chronic discomfort. Optimizing pain management techniques stands as a pivotal factor in enhancing the well-being and overall quality of life for patients. This comprehensive review discusses various pain conditions encountered after head and neck cancer and explores a multidimensional approach to pain management. The review highlights the significance of incorporating multimodal analgesia, physical therapy, psychological support, palliative care, and emerging techniques including nerve blocks to achieve efficacious pain control. Such an endeavor necessitates cooperation among head and neck surgeons, radiotherapists, and pain specialists.

## 1. Context

The intricacies posed by head and neck cancer render the task of pain management multifaceted, owing to its intricate anatomical site and potential interference with pivotal functions like speech, swallowing, and respiration. Pain stemming from head and neck cancer surgeries emerges from diverse sources, encompassing surgical trauma, nerve damage, inflammation, and tissue scarring. Furthermore, pain induced by radiotherapy and complications such as sternocleidomastoid (SCM) muscle spasms and the perplexing First Bite Syndrome further contribute to the pain burden experienced by these patients.

## 2. Search Strategy

To comprehensively assess current practices and identify potential areas for improvement, we conducted a systematic search of the PubMed bibliographic database using Medical Subject Headings (MeSH) keywords: "Neck pain" [Mesh: D054300], "cancer pain" [Mesh: D006175], "pain management" [Mesh: D009421], "chronic pain" [Mesh: D008288], and "head and neck neoplasms" [Mesh: D005200]. This search identified studies investigating the prevalence and characteristics of pain in head and neck cancer patients, as well as the efficacy of various pain management strategies, including multimodal analgesia and interventional procedures. The findings from these studies were critically appraised and synthesized to provide a comprehensive narrative of the current state of knowledge on optimizing pain management in head and neck cancer.

## 3. Pain Conditions and Management Strategies

### 3.1. Surgical Site Pain

Persistent pain at the surgical site stands as a prevailing concern subsequent to head and neck cancer surgeries. A recommended approach involves nonsteroidal anti-inflammatory drugs (NSAIDs), supplementary analgesics, opioids, and local anesthetics within a comprehensive analgesic strategy. The use of regional anesthesia techniques, including cervical epidural blockade and local anesthetic infiltration, can provide targeted pain relief and reduce opioid requirements (1, 2). Moreover, physical therapy interventions, including exercises and manual therapy, can improve the range of motion and functional recovery (3).

### 3.2. Shoulder Pain after Radical Neck Surgery

One of the most common chronic pain syndromes following radical neck surgery is shoulder pain. The surgical excision of cervical lymph nodes and the concomitant disruption of the spinal accessory nerve culminate in shoulder dysfunction and pain (3). The first line of management typically involves NSAIDs and physical therapy such as scapular muscle exercises (4). These exercises should focus on restoring range of motion, strengthening weakened muscles, and improving postural alignment. However, when conservative methods are unsatisfactory, interventional pain procedures can be considered. Spinal accessory nerve block (SANB) has shown efficacy in providing pain relief when the trapezius muscle is the source of pain (5). In instances resistant to conventional measures including trigger point injections (6), the application of ipsilateral stellate ganglion block (SGB), impeding sympathetic outflow to the upper extremity, may provide pain relief and improve shoulder function (7).

### 3.3. Glossopharyngeal Neuralgia

Glossopharyngeal neuralgia is a rare but excruciating pain condition characterized by severe, lancinating pain in the oropharynx, ear, and base of the tongue. It can occur following surgical interventions such as glossectomy. First-line pharmacological management involves the use of antiepileptic drugs, such as carbamazepine and gabapentin, to reduce neuronal excitability and dampen pain signals (8). However, in cases refractory to medical therapy, glossopharyngeal nerve block can provide targeted pain relief (9). This procedure involves the percutaneous injection of a local anesthetic near the glossopharyngeal nerve to interrupt pain transmission. In case of temporary pain relief, neuromodulation of the nerve with pulsed radiofrequency (10) or possibly radiosurgery with Gamma knife can be considered (11).

### 3.4. First Bite Syndrome

First Bite Syndrome, a sporadic yet distressing occurrence, may manifest after parotidectomy, carotid endarterectomy, or other surgeries that engage the infratemporal fossa. Patients complain of acute pain within the parotid region upon the initial bites of food. While a complete comprehension of the underlying pathophysiology remains elusive, it is postulated to entail damage or denervation of sympathetic nerve fibers and parasympathetic hyperactivation. Initial interventions typically encompass a soft diet and analgesic usage. Pharmacological options encompass anticholinergic agents, tricyclic antidepressants, and anticonvulsants (12). Nevertheless, when conservative approaches fail to provide adequate relief, minimally invasive procedures can be considered. Intra-parotid Botulinum toxin A causes parasympathetic nerve paralysis of the parotid gland and would minimize salivation and decrease first bite syndrome (13). Sporadic reports are also available regarding the therapeutic potential for blockade or ablation of the branches of the trigeminal nerve in refractory cases (14). It has been postulated that this treatment may disrupt somatic sensory input from the parotid carried by the auriculotemporal nerve or lesion the otic ganglion resulting in decreased parasympathetic hyperactivation.

### 3.5. Trismus

Trismus is a common complication of head and neck cancer treatment, particularly in scenarios involving oral cavity and jaw surgery and after radiotherapy. The ability to eat, speak, and maintain oral hygiene is significantly hampered. Approaches to management encompass physical therapy entailing jaw exercises (15) and low-level laser (16). Pentoxifylline has been suggested to treat radiation-induced fibrosis and trismus (17, 18). Botulinum toxin injections into the masseter muscle may decrease pain and spasticity, but has resulted in improvement of trismus (19). The use of mandibular nerve block for trismus in head and neck cancer patients has not been investigated, but due to its effectiveness in similar scenarios, it deserves further investigation (20, 21).

### 3.6. Sternocleidomastoid Spasm and Fibrosis

Sternocleidomastoid (SCM) muscle spasms, which may occur after head and neck surgery, particularly after radiotherapy, elicit considerable pain and functional impairment (22). The implementation of non-pharmacological measures such as heat therapy and physical therapy techniques can yield benefits (23). Furthermore, the introduction of botulinum toxin (Botox) into the affected muscle exhibits the potential to instigate muscle relaxation by obstructing acetylcholine release, thereby alleviating pain (24).

### 3.7. Radiotherapy-Induced Mucositis and Irritating Pain

Radiotherapy-induced oral pain and mucositis present significant challenges in the management of cancer patients undergoing radiation treatment. This condition is characterized by inflammation and ulceration of the oral mucosa, leading to severe discomfort and potential complications such as difficulty in eating, speaking, and an increased risk of infection. The mechanisms underlying radiotherapy-induced oral mucositis involve direct damage to the epithelial cells lining the oral cavity, as well as inflammatory responses mediated by cytokines and reactive oxygen species (25). Treatment guidelines aim to provide nutritional support while reducing pain, inflammation, hemorrhaging, and oral microbial contamination (26). Effective management strategies include preventive measures such as oral care protocols, pharmacological interventions including analgesics and anti-inflammatory agents, and supportive therapies like cryotherapy and low-level laser therapy (25).

After ensuring oral hygiene, pharmacological interventions are essential components of management. Topical agents like mucosal coating agents (e.g., sucralfate) and anesthetics (e.g., lidocaine mouthwash) possibly provide localized relief and promote healing. Recently, the use of Doxepine or diphenhydramine-lidocaine-antacid mouthwash has been suggested (27). Oral NSAIDs may be required. Additionally, the use of anti-inflammatory agents like corticosteroids or possibly the radioprotective agent amifostine may help reduce mucosal inflammation (28). Supportive therapies such as cryotherapy, where ice chips are applied to the oral mucosa during radiation sessions, can also mitigate mucosal damage. Low-level laser therapy has shown promise in accelerating mucosal healing and reducing pain (25). Furthermore, nutritional support through dietary counseling and supplementation with vitamins and minerals may aid in maintaining oral health and supporting tissue repair. Close collaboration between ENT surgeons, pain physicians, oncologists, and dental specialists is essential to tailor treatment strategies to individual patient needs and optimize outcomes in this challenging clinical scenario.

### 3.8. Radiotherapy- and Chemotherapy-Induced Neuropathic Pain

Neuropathic pain poses a significant challenge in the management of head and neck cancer patients, often leading to debilitating symptoms and decreased quality of life (29). This form of neuropathic pain arises due to damage to peripheral nerves or central nervous system structures as a result of cancer treatment. Treatment strategies typically involve a combination of pharmacological interventions, including tricyclic antidepressants, anticonvulsants (such as gabapentin or pregabalin), ketamine, methadone, and serotonin-noradrenaline reuptake inhibitors, which target neuropathic pain mechanisms (30). In addition to medications, interventional procedures like nerve blocks or neurolytic techniques may offer localized pain relief (31). Neuromodulation techniques such as peripheral nerve field stimulation (32), spinal cord stimulation (SCS) (33), and recently transauricular vagal nerve stimulation (taVNS) (34) have been suggested to relieve neuropathic pain and improve the quality of life in these patients. There are few studies supporting the use of sympathetic blocks such as Sphenopalatine and stellate ganglion blocks in head and neck cancer pain management (35, 36). Patient-centered care, tailored to individual needs and preferences, is paramount in optimizing outcomes and improving the quality of life in these patients.

### 3.9. Other Chronic Pain Syndromes

In addition to the aforementioned pain conditions, head and neck cancer patients may experience a variety of other chronic pain syndromes, including oromandibular dystonia and pain stemming from radiation-induced fibrosis (37). The management of these conditions necessitates a multimodal approach. Pharmacological interventions, like analgesics (e.g., gabapentin, amitriptyline), topical agents (e.g., lidocaine patches), and even opioids, are contingent on the specific nature of the pain. Physical therapy, low-level laser application, and interventional pain procedures, namely Botulinum toxin injection, can also play a significant role in improving patients' pain control and overall well-being (19, 37, 38).

## 4. The Role of Psychological Support and Palliative Care

The psychological component of chronic pain within head and neck cancer patients warrants consideration (39). Integrating psychological support, such as cognitive-behavioral therapy and mindfulness-based techniques, can help address psychological factors and enhance pain management outcomes (40-42). Palliative care measures, including symptom management, nutritional support, speech therapy, and social support, stand as pivotal agents in enhancing the quality of life for those confronting advanced head and neck cancer (43, 44).

## 5. Emerging Techniques and Future Directions

The field of pain management is continually evolving, with emerging techniques offering potential benefits for head and neck cancer patients. Neurostimulation, radiofrequency ablation, and neuromodulation are being explored as innovative approaches. Moreover, ongoing research into novel pharmacological agents, precision-targeted drug delivery systems, and individualized pain management strategies bear the promise of ushering in a more sanguine future.

## 6. Conclusions

Effective pain management stands as an imperative facet in optimizing outcomes and improving the quality of life for head and neck cancer patients. The collaborative engagement between head and neck surgeons, radiation oncologists, and pain specialists emerges as a pivotal force in identifying and tending to the myriad pain conditions that may emerge post-surgery and radiotherapy. Referral to a pain specialist should be considered for patients with persistent or severe pain, particularly when conservative measures have been unsuccessful. Multimodal approaches, encompassing pharmacological interventions, physical therapy, and interventional pain procedures, individually tailored to distinct pain conditions, stand as key enablers of pain alleviation, functional improvement, and overall well-being for patients.
